# TEFM is a potent stimulator of mitochondrial transcription elongation *in vitro*

**DOI:** 10.1093/nar/gkv105

**Published:** 2015-02-17

**Authors:** Viktor Posse, Saba Shahzad, Maria Falkenberg, B. Martin Hällberg, Claes M. Gustafsson

**Affiliations:** 1 Department of Medical Biochemistry and Cell Biology, University of Gothenburg, SE-40530 Gothenburg, Sweden; 2 Department of Cell and Molecular Biology, Karolinska Institutet, SE-17177 Stockholm, Sweden; 3 Röntgen-Ångström-Cluster, Karolinska Institutet Outstation, Centre for Structural Systems Biology, DESY-Campus, D-22603 Hamburg, Germany; 4 European Molecular Biology Laboratory, Hamburg Unit, D-22603 Hamburg, Germany

## Abstract

A single-subunit RNA polymerase, POLRMT, transcribes the mitochondrial genome in human cells. Recently, a factor termed as the mitochondrial transcription elongation factor, TEFM, was shown to stimulate transcription elongation *in vivo*, but its effect *in vitro* was relatively modest. In the current work, we have isolated active TEFM in recombinant form and used a reconstituted *in vitro* transcription system to characterize its activities. We show that TEFM strongly promotes POLRMT processivity as it dramatically stimulates the formation of longer transcripts. TEFM also abolishes premature transcription termination at conserved sequence block II, an event that has been linked to primer formation during initiation of mtDNA synthesis. We show that POLRMT pauses at a wide range of sites in a given DNA sequence. In the absence of TEFM, this leads to termination; however, the presence of TEFM abolishes this effect and aids POLRMT in continuation of transcription. Further, we show that TEFM substantially increases the POLRMT affinity to an elongation-like DNA:RNA template. In combination with previously published *in vivo* observations, our data establish TEFM as an essential component of the mitochondrial transcription machinery.

## INTRODUCTION

The human mitochondrial genome (mtDNA) is a circular DNA molecule of 16.6 kb that encodes 22 tRNAs, 2 rRNAs and 13 proteins required for oxidative phosphorylation. Transcription of the genome is initiated from two regulatory sites in the control region of mtDNA, the heavy- and light-strand promoters (HSP1 and LSP). These promoters direct transcription in opposite directions and generate polycistronic, near full genome-sized transcripts that are processed to produce individual RNA molecules. Transcription initiated at LSP also provides primers for the replication of mtDNA (1). During transcription of the conserved sequence block (CSB) region located downstream from LSP, a G-quadruplex structure is formed in the nascent RNA, which stimulates transcription termination and formation of shorter transcripts that may be used for the initiation of H-strand mtDNA synthesis (2–5).

Transcription in mammalian mitochondria is carried out by the mitochondrial RNA polymerase, POLRMT (1). POLRMT is a single-subunit polymerase that contains a catalytic C-terminal domain (CTD) and an N-terminal domain (NTD) that are similar in sequence and structure to the T7 RNA polymerase (T7 RNAP) (6). POLRMT also contains an N-terminal extension (NTE) that is not present in T7 RNAP (7). Furthermore, in contrast to T7 RNAP, POLRMT cannot initiate promoter-dependent transcription on its own, as it needs two additional transcription factors, TFAM and TFB2M (1,8). These two factors are needed for the recruitment of POLRMT (9,10) and initial unwinding of the double-stranded promoter region (11), but they are not required for the transcription of a single-stranded DNA template or a template with a single-stranded DNA bubble covering the transcription start site (12).

**Figure 1.**
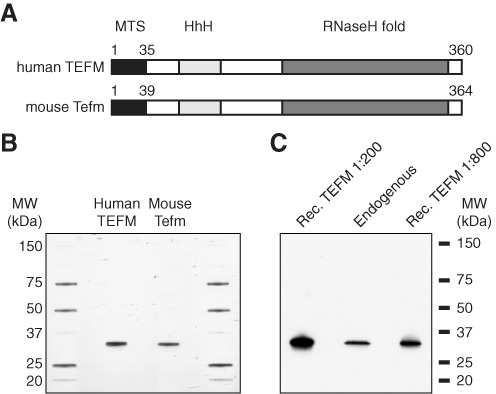
Production of recombinant human TEFM and mouse Tefm. (**A**) Schematic representation of human TEFM and mouse Tefm. MTS (mitochondrial targeting signal), HhH (helix–hairpin–helix fold) and RnaseH fold (RuvC type) are indicated. (**B**) SDS-PAGE of recombinant human TEFM (amino acids 36–360) and mouse Tefm (amino acids 40–364). Both proteins run slightly below the expected size of 37.6 kDa. Molecular weight markers (kDa) are found in the first and last lanes. (**C**) Immunoblotting of recombinant human TEFM and endogenous TEFM from HeLa cells. Molecular weight marker is indicated.

POLRMT produces near genomic-length transcripts *in vivo;* therefore, the enzyme has to be processive. TEFM (mitochondrial transcription elongation factor) was identified based on its sequence similarities to known transcription elongation factors (13). The protein contains two conserved fold domains: a RuvC-type RnaseH-fold domain and a helix–hairpin–helix (HhH) motif that is found in other transcription-related DNA-binding proteins (Figure 1A). TEFM physically interacts with POLRMT, and RNA knockdown of TEFM *in vivo* leads to a decrease in promoter distal transcripts, demonstrating that TEFM is required for transcription elongation (13). The effect of TEFM *in vitro* was limited, with a modest stimulatory effect on POLRMT-dependent transcription on single-stranded DNA (ssDNA) and tailed, double-stranded DNA (dsDNA) templates.

We found the previous study and identification of TEFM intriguing (13) and decided to characterize how this protein influences the core transcription machinery using a reconstituted *in vitro* transcription system (8,14). As demonstrated below, we have established conditions that allow us to purify highly active TEFM in its recombinant form. In our assays, TEFM has a dramatic stimulatory effect on transcription elongation *in vitro*, and together with the previously published *in vivo* data, our findings help establish TEFM as an essential component of the mitochondrial transcription machinery.

## MATERIALS AND METHODS

### Protein expression, purification and analysis

N-terminally His_6_-tagged human TEFM (residues 36–360; UniProt: Q96QE5) and mouse Tefm (residues 40–364; UniProt: Q5SSK3) were expressed recombinantly in KRX cells (Promega). Expression in Terrific broth supplemented with 8 g/l glycerol was induced at 18°C by the addition of 0.2% rhamnose and 0.5 mM isopropyl β-D-1-thiogalactopyranoside (IPTG). Induction was maintained for 18 h. Harvested cells were resuspended in lysis buffer (100 mM HEPES pH 8.0, 0.5 M NaCl, 10% glycerol, 10 mM imidazole and 0.5 mM Tris (2-carboxyethyl) phosphine (TCEP)) and lysed in a Microfluidizer M110-L (Microfluidics), followed by clarification. Initial purification was performed with a 5 ml HiTrap Ni-IMAC column developed with a gradient from 40 to 500 mM imidazole in the lysis buffer. The His_6_-tag fusion elements were excised through incubation with His-tagged tobacco etch virus protease (1:30 protease:protein ratio) for 18 h at 4°C followed by a repeated IMAC purification, this time collecting the flow through. The flow through was bound on a 5 ml HiTrap Heparin column. The heparin column was developed with a linear NaCl gradient (150 mM–1 M) in lysis buffer. TEFM eluted at ∼0.6 M NaCl. The TEFM peak fractions were concentrated and further purified on a Sephacryl S-200 HR 16/60 gel filtration column equilibrated in 20 mM HEPES pH 7.5, 300 mM NaCl, 10% glycerol and 2 mM TCEP. The purified human and mouse TEFM samples were concentrated to 4 and 8 mg/ml, respectively, and were aliquoted and stored at −80°C. All columns used were from GE Healthcare, Uppsala, Sweden. The human POLRMT was expressed as a TEV protease cleavable MBP fusion protein in *Escherichia coli*, mouse Polrmt and TFAM and TFB2M, mouse and human proteins, were expressed and purified as previously described (10,14,15). Recombinant and endogenous (HeLa) TEFM was analysed with immunoblotting using an anti-TEFM antibody (HPA023788, Sigma–Aldrich).

### MicroScale thermophoresis

The interaction affinities of POLRMT to TEFM and POLRMT to a DNA:RNA template were studied using microscale thermophoresis (MST), as implemented in the Monolith NT.115 instrument (NanoTemper Technologies GmbH, Munich, Germany). Standard capillaries with 80% LED power and 40% MST power for 30 s at 22°C were used for the POLRMT–TEFM experiments and standard capillaries with 80% LED power and 60% MST power for 15 s at 22°C for the POLRMT/TEFM–DNA:RNA experiments. For POLRMT–TEFM interactions, TEFM was amine-labeled with 2-fold excess of NT-547 dye (Nanotemper) using the manufacturer's protocol. For the DNA:RNA interaction, an 18-mer 5′ Alexa488-labeled DNA (5′-GGG AAT GCA TGG CGC GGC-3′) hybridized to a 12-mer of RNA (5′-UUU UGC CGC GCC-3′) was used (Eurofins Operon). Unlabeled POLRMT was diluted in twelve 2-fold dilution series starting from 2750 and 1000 nM (final concentration) for TEFM and DNA:RNA interactions. Twelve 2-fold dilution series starting at 13.7 μM of TEFM were used for the TEFM–DNA:RNA experiment. 50 nM of labeled TEFM or 15 nM of the DNA:RNA template (final concentrations) was added to the dilutions. The complexes were incubated for 10 min at room temperature in the final buffer containing 25 mM Tris–HCl pH 8.0, 100 mM NaCl, 10 mM MgCl_2_, 10% glycerol and 1 mM dithiothreitol (DTT). For the DNA:RNA interaction the final buffer also contained 500 μM ATP and 0.05% Tween-20, the interaction was measured in the presence or absence of 2000 nM unlabeled TEFM. Each experiment was done in triplicate. Temperature jump data were used for TEFM interactions whereas combined thermophoresis and temperature jump data were used for DNA:RNA experiments. Nanotemper analysis software was used for *K*_d_, bound state, and unbound state calculations. Baseline-corrected, normalized and scaled change in fluorescence was plotted against the log of the range of the POLRMT or TEFM concentrations as indicated.

### Template preparation

The templates used for transcription were either linearized plasmid vectors as previously reported (14,15) or short ligase chain reaction (LCR) produced templates (for AP and 8-Oxo-dG templates). For the human system, a plasmid template in the form of a pUC18 vector with a human mitochondrial promoter region insert was used (human mtDNA 1–477 for LSP and 499–742 for HSP, cloned between BamHI and HindIII restriction sites). For the mouse system a pCR4-TOPO vector with a mouse LSP region insert was used (mouse mtDNA 15 941–16 260). Alternative linearization cleavage sites were used to generate templates for run-off transcription of desired lengths. To generate run-off transcripts of about 3000 nts, the human templates were cleaved with HindIII (for LSP) or BamHI (for HSP). To generate run-off transcripts of about 400 nts, BamHI (for LSP) and NdeI (for HSP) were used. To generate a 4500 nts long run-off template for mouse LSP transcription, NotI was used. For the T7 template, the pBluescript vector with a CSB region insert (human mtDNA 1–393) cloned downstream of the T7 promoter was used (16). The LSP PCR 2000 nts run-off template was produced using PCR on human mtDNA with primer pair 5′-CCT ATA TTA CGG ATC ATT TCT CTA C and 5′-GTA GTA TGG GAG TGG GAG G followed by PCR purification (Quiagen). The LCR templates were produced using 2 oligonucleotide pairs and a bridging oligo. LSP promoter oligos were: forward 5′-TGA TGA GAT TAG TAG TAT GGG AGT GGG AGG GGA AAA TAA TGT GTT AGT TGG GGG GTG ACT GTT AAA AGT GCA TAC CGC CA and reversed (with 5′ phosphate) 5′-TGG CGG TAT GCA CTT TTA ACA GTC ACC CCC CAA CTA ACA CAT TAT TTT CCC CTC CCA CTC CCA TAC TAC TAA TCT CAT CA. LSP downstream oligos were: forward (with 5′ phosphate) 5′-AAA GAT AAA ATT TGA AAT CTG GTT AGG CTG GTG TTA GGG TTC TTT GTT TCT GGG GTT TGG CAG AG and reversed 5′-CTC TGC CAA ACC CCA XAA ACA AAG AAC CCT AAC ACC AGC CTA ACC AGA TTT CAA ATT TTA TCT TT where X indicates the WT dG, the 8-Oxo-dG (Thermo Scientific) or dSpacer modification (Eurofins Operon), as bridging oligo GAC TGT TAA AAG TGC ATA CCG CCA AAA GAT AAA ATT TGA AAT CTG G was used. The templates were produced mixing 10 pmol of all template oligos with 1 pmol of bridging oligo in a 50 μl Taq ligase (New England Biolabs) reaction followed by LCR for 50 cycles with 94°C for 15 s, 40°C for 30 s 65°C for 1 min.

### *In vitro* transcription

Standard transcription reactions contained 25 mM Tris–HCl pH 8.0, 10 mM MgCl_2_, 64 mM NaCl, 100 μg/ml bovine serum albumin (BSA), 1 mM DTT, 400 μM ATP, 150 μM GTP, 150 μM CTP, 10 μM UTP, 0.02 μM α-^32^P UTP (3000 Ci/mmol), 4 U RNase inhibitor Murine (New England Biolabs), 3.6 nM of linearized plasmid template or 8 nM of LCR template, 20 nM of POLRMT for plasmid or 40 nM for LCR template, 60 nM of TFB2M for plasmid or 80 nM for LCR template, and the TEFM concentrations indicated. The concentrations of TFAM varied between 100 and 180 nM for plasmid templates and 23 nM for LCR templates. A too high ratio of TFAM relative to DNA will inhibit transcription (17). TFAM levels must therefore be adjusted depending on the length and concentration of DNA templates used. The volume of standard reactions was 25 μl. The solutions were mixed on ice, with the protein added last, and the reactions were initiated by moving the mixtures to 32°C, where they were incubated for 30 min unless otherwise stated. For time-point experiments, the reactions were performed in master mixes, and 25 μl aliquots were taken at the indicated time points. For transcription with T7 RNA Polymerase (T7 RNAP), a 25 μl reaction contained 1× RNAP buffer (New England Biolabs), 100 μM ATP, 100 μM GTP, 100 μM CTP, 10 μM UTP, 0.02 μM α-^32^P UTP (3000 Ci/mmol), 4 U RNase inhibitor Murine (New England Biolabs), 3.6 nM of linearized plasmid template, and 2 U T7 RNAP (New England Biolabs). The reactions were performed at 37°C for 5 min. All reactions were stopped by the addition of a stop buffer (10 mM Tris–HCl pH 8.0, 0.2 M NaCl, 1 mM ethylenediaminetetraacetic acid (EDTA), and 100 μg/ml proteinase K), followed by incubation at 42°C for 45 min. Transcription products were purified with phenol extraction and ethanol precipitation, and the pellets were dissolved in 20 μl gel-loading buffer (98% formamide, 10 mM EDTA, 0.025% xylene cyanol FF, and 0.025% bromophenol blue) and heated at 95°C for 3 min. The samples were analyzed on 4–6% denaturing polyacrylamide gels (1× Tris/Borate/EDTA (TBE)-buffer) and 7 M urea) followed by exposure on photo film or Phosphorimager. The pulse-chase experiment was performed as before (2), with the following changes: the transcription reaction was initiated as above in the presence or absence of 40 nM TEFM, and after 3 min the radiolabeling was stopped by addition of 0.5 μl 100 mM UTP per 25 μl reaction. TEFM was added to a subset of the samples lacking TEFM from the start, and time points were collected as above. Quantifications of transcripts were performed using the program Multi Gauge with images generated from a Phosphorimager. All results have proved to be reproducible in at least three experiments, and a representative figure for each experiment was chosen.

### DNase I footprinting

The footprinting template was produced by PCR on the pUC18 human LSP vector using the primer pair 5′-GCA CTT AAA CAC ATC TCT GCC AAA CCC C (forward) and 5′-GTA AAA CGA CGG CCA GTG CCA AGC (reversed, found in vector). The forward primer was labeled with PNK enzyme (New England Biolabs) and γ-^32^P ATP (3000 Ci/mmol). The footprint reaction was performed in 20 μl containing 25 mM Tris–HCl pH 8.0, 10 mM MgCl_2_, 1 mM ATP, 100 μg/ml BSA, 1 mM DTT, 50 mM NaCl, labeled template (22 500 cpm), and proteins as indicated (1 pmol TFAM, 1 pmol POLRMT, 2 pmol TFB2M and 2 pmol TEFM). The mixture was incubated for 20 min at room temperature followed by the addition of 2 μl of 50 mU/μl DNase I diluted in 2.5× DNase I buffer with MgCl_2_ (Thermo Scientific). The DNase I reaction was stopped after 2 min by the addition of 20 μl stop buffer (200 mM NaCl, 20 mM EDTA, 1% sodium dodecyl sulphate (SDS) and 100 μg/ml yeast tRNA (Ambion)) directly followed by incubation on ice. The DNA fragments were recovered with phenol extraction and ethanol precipitation and analyzed on an 8% denaturing polyacrylamide sequencing gel (1× TBE and 7 M urea).

## RESULTS

### Recombinant TEFM interacts with POLRMT and drastically decreases pre-termination events

To study transcription elongation *in vitro*, we cloned, expressed, and purified TEFM, and its mouse homologue denoted Tefm (Figure 1A and B). The recombinant proteins lacked the mitochondrial targeting signals (MTS), which had been predicted using MITOPROT (http://ihg.gsf.de/ihg/mitoprot.html). The MITOPROT-derived cleavage sites were examined with secondary structure predictions to minimize chances of interruption of structural segments. The recombinant TEFM and Tefm proteins used in the study are both 234 residues long with a molecular weight of 37.6 kDa. The recombinant TEFM protein migrated somewhat faster than expected; however, immunoblotting analysis revealed that its apparent molecular weight was nearly identical to that of the endogenous TEFM protein (Figure 1C). To investigate the native quaternary structure of TEFM, the protein was analysed with analytic gel filtration analysis. TEFM migrated at an apparent molecular weight of 82 kDa (Supplementary Figure S1), which suggest that it is a dimer in solution.

**Figure 2.**
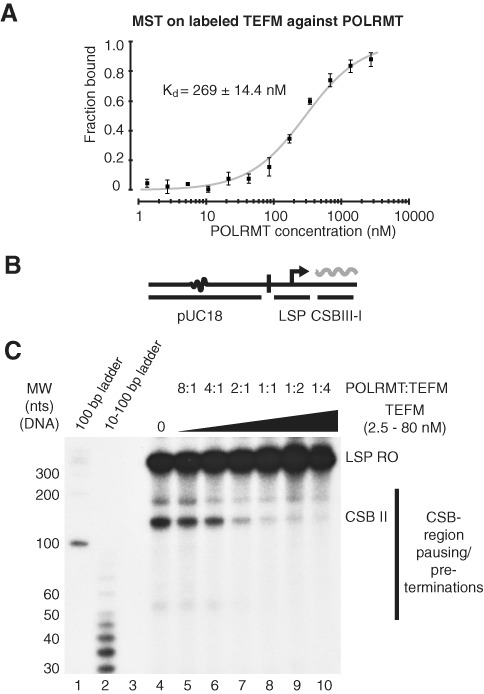
TEFM interacts directly with POLRMT to increase the polymerase processivity. (**A**) Microscale thermophoresis (MST) on labeled TEFM against POLRMT as described in materials and methods. The estimated fraction bound based on temperature jump data was plotted against POLRMT concentration giving a *K*_d_ value of 269 ± 14.4 nM. The experiment was performed in triplicate, and error bars show standard deviation. (**B**) Cartoon of the template used, the pUC18 vector with a mitochondrial LSP region insert including the CSB region, producing a run-off transcript of approximately 400 nts. (**C**) *In vitro* transcription with titration of TEFM. Lanes 1 and 2 contain DNA ladders (NEB 100 bp ladder and Affymetrix low molecular weight 10–100 nt ladder respectively; lane 3 is empty, and lanes 4–10 contain transcription reactions). Lane 4 has no TEFM added followed by lanes with TEFM in increasing concentrations (2.5, 5, 10, 20, 40, 80 nM in lanes 5–10, respectively). POLRMT to TEFM ratio, CSB region pre-terminations, CSB II, as well as run-off (RO) transcripts are indicated.

To examine the activity of our recombinant TEFM and to confirm the previously reported interactions between TEFM and POLRMT (13), we used microscale thermophoresis (MST). Using labeled TEFM and different concentrations of POLRMT, we observed a clear binding between the two components, and the *K*_d_ was determined as 269 ± 14.4 nM (Figure 2A). In addition to confirming the interaction between POLRMT and TEFM, this result also shows that the interaction is direct and present in solution in the absence of DNA, RNA and additional protein factors.

To investigate the effects on the core mitochondrial transcription machinery *in vitro*, we added TEFM in increasing concentrations to transcription reactions containing POLRMT, TFAM, TFB2M and a linearized DNA template containing LSP. The template was expected to generate a run-off product of approximately 400 nucleotides (nts) (Figure 2B). We observed a modest increase in run-off transcription with increasing concentration of TEFM (Figure 2C, compare LSP RO in lane 4 with lanes 5–10), consistent with a stimulatory effect on transcription elongation. The template used also contained the conserved sequence block (CSB) region. We have previously demonstrated that a large fraction (up to 65%) of LSP transcription events are prematurely terminated at CSB II due to G-quadruplex formation in nascent RNA (2,5). In the absence of TEFM, we could, indeed, observe pre-termination of transcripts at CSB II (Figure 2C, lane 4). However, the addition of TEFM caused a dose-dependent disappearance of these pre-terminated transcripts (Figure 2C, lanes 5–10). Apart from the abundant CSB II pre-terminated transcript, we could also observe other, less abundant transcripts that were terminated in the CSB region and decreased upon the addition of TEFM. We conclude that TEFM stimulates transcription elongation and prevents premature termination of transcription in the CSB region of mtDNA. We have previously demonstrated that the activities of the mitochondrial transcription machinery are sensitive to salt conditions (18). To analyze how salt affects the action of TEFM, we monitored transcription using varying concentrations of NaCl and MgCl_2_. Changes in salt concentrations affected the overall transcription levels, but did not affect the function of TEFM (Supplementary Figure S2A and B).

### TEFM is essential for POLRMT to generate long transcripts

**Figure 3.**
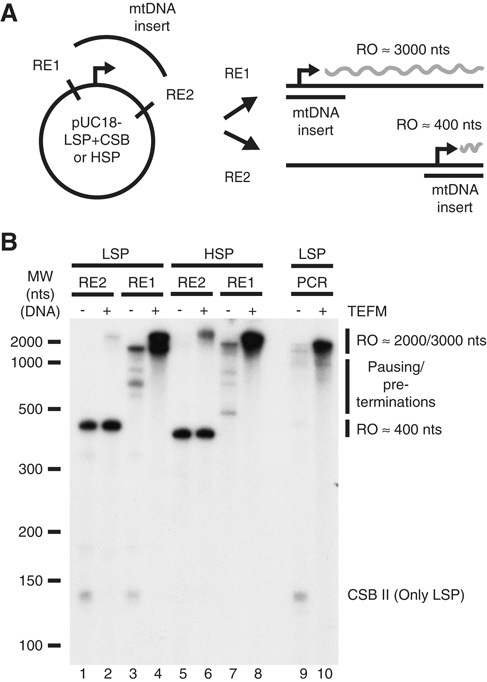
TEFM strongly promotes POLRMT processivity on longer templates. (**A**) Cartoon of the templates used, produced from pUC18 containing an HSP or LSP insert (mtDNA insert). Note that only the LSP template contains the CSB region. To create run-off products of different lengths, the plasmids were cleaved with different restriction enzymes (RE) as specified in materials and methods. (**B**) *In vitro* transcription from LSP (lanes 1–4 and 9–10) or HSP (lanes 5–8) templates. These templates generate transcripts of about 400 nts (lanes 1–2 and 5–6), 3000 nts (lanes 3–4 and 7–8) as shown in panel (A), or 2000 nts (lanes 9–10, LSP PCR fragment template). TEFM was added at 40 nM where indicated. Note that only the LSP templates contain the CSB region, explaining the indicated CSB II-terminated bands present in LSP templates in the absence of TEFM.

*In vivo*, the mitochondrial transcription machinery has to produce transcripts corresponding to almost the entire 16.6 kb mtDNA molecule. To investigate how TEFM influences POLRMT transcription of longer stretches of DNA, we used a LSP containing template that was cleaved to generate run-off transcripts of 400 or 3000 nts. To verify that observed effects were not promoter-specific, we generated HSP1 templates of similar length (Figure 3A). We monitored transcription from the four templates in the presence and absence of TEFM (2:1 ratio of TEFM:POLRMT). On the shorter run-off template, TEFM had a small stimulatory effect on full-length LSP transcription consistent with the results shown in Figure 2C; however, no apparent effect could be observed on transcription initiating from HSP1 (Figure 3B, compare lanes 1–2 and 5–6). The stronger effect on full-length LSP transcripts could be explained by TEFM's ability to decrease premature transcription termination at CSB II and, thus, promote run-off transcription (see Figure 2C), since there are no transcription termination signals of similar strength immediately downstream of HSP1. When examining the longer run-off templates in the absence of TEFM, the full-length run-off transcripts represented only a small fraction of the total transcript levels, with clear pre-terminations not only in the CSB region (Figure 3B, lane 3), but also in the vector part of the LSP and the HSP1 templates (Figure 3B, lanes 3 and 7). The addition of TEFM led to a dramatic change in the ratio between full-length transcripts and shorter transcripts. The run-off product was now by far the most dominant transcript, both for LSP and HSP1, reaching much higher levels than in the absence of TEFM. Additionally, as seen with the CSB II transcript, the levels of all pre-maturely terminated transcripts were strongly decreased (Figure 3B, lanes 4 and 8). Our results demonstrate that TFAM, TFB2M and POLRMT are insufficient for successful transcription of longer stretches of DNA, producing only low levels of run-off transcripts. Addition of TEFM shifted the balance, producing high levels of run-off transcripts and almost no pre-terminated products. The templates used in our experiments contained long regions of plasmid DNA, but we obtained similar results when we used a PCR fragment containing the LSP promoter followed by a 2000 bp long native mtDNA sequence (Figure 3B, lanes 9–10).

### TEFM stabilizes POLRMT-interactions with DNA and reduces transcription termination

**Figure 4.**
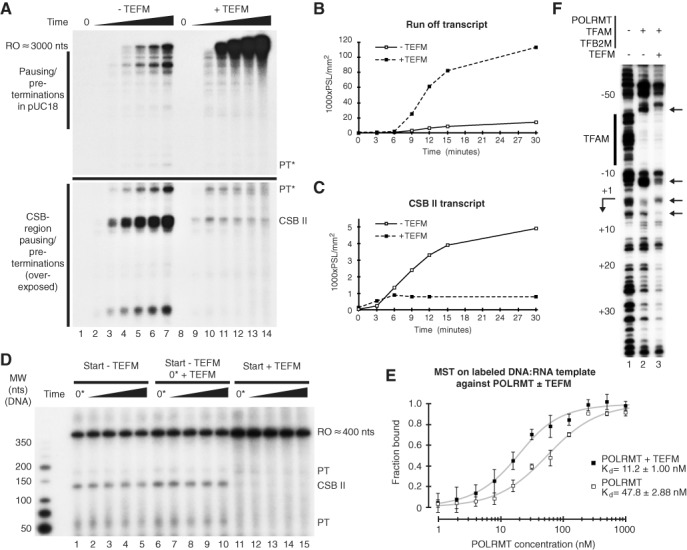
TEFM prevents termination at transcription pause sites and increases POLRMT affinity to DNA. (**A**) *In vitro* transcription from LSP on the 3000 nts run-off template (Figure 3A) at different time points (0, 3, 6, 9, 12, 15 and 30 min) in the absence (lanes 1–7) or presence (lanes 8–14) of 40 nM TEFM. The upper part of the figure is underexposed, and the bottom part is overexposed to compensate for a difference in labeling due to transcript length. For comparison, the pre-terminated transcript labeled PT* is shown in both parts. Full figures of both exposures are found in Supplementary Figure S3A and B. CSB region transcripts as well as run-off transcripts are indicated. (**B**) Quantification of run-off transcripts in panel (A). White squares with full black lines indicate samples in the absence of TEFM, and black squares with dotted lines indicate samples in the presence of TEFM. The transcript levels are measured in photostimulated luminescence per area (PSL/mm^2^) and the time in minutes. (**C**) The same quantification as in panel (B) but for the CSB II transcript. (**D**) Pulse-chase experiment on the 400 nts run-off LSP template. Transcription was initiated in the absence (lanes 1 and 6) or presence of 40 nM of TEFM (lane 11). After 3 min incubation, an excess of cold UTP was added to stop labeling. At this time point (0*), one of the reaction mixtures lacking TEFM was supplemented with 40 nM of TEFM (lanes 6–10). The reactions were then allowed to progress and samples were taken for analysis after 2.5, 5, 10 and 30 min. Transcripts prematurely terminated in the CSB region (PT and CSB II) as well as run-off transcripts are indicated. LMW marker (New England Biolabs) is indicated. (**E**) Microscale thermophoresis on an 18-mer 5′ Alexa488 labeled DNA hybridized to 8 nts of a 12-mer of RNA against POLRMT in the presence of ATP and in the presence or absence of TEFM. In the presence of ATP, the template allows for one nt incorporation before pausing. The estimated fraction bound based on combined thermophoresis and temperature jump data was plotted against POLRMT in the absence, white squares, or in the presence, filled black squares, of 2000 nM TEFM. The *K*_d_ of the interactions were determined as 47.8 ± 2.88 nM in the absence and 11.2 ± 1.00 nM in the presence of TEFM. The experiments were performed in triplicate and error bars show standard deviation. (**F**) DNase I footprinting on an LSP PCR template. Lane 1 is a no-protein control whereas lane 2 contains the initiation machinery (POLRMT, TFAM and TFB2M) and lane 3 contains the initiation machinery complemented with TEFM. Arrows indicate differences between samples in the absence or presence of TEFM (lanes 2 and 3, respectively). Relative positions to LSP +1 as well as the TFAM binding site are indicated.

To further elucidate the function of TEFM, we performed a time-point experiment on the long LSP run-off template (Figure 3A). As observed before, in the absence of TEFM, the full-length run-off transcripts represented only a fraction of the total transcripts, with clear pre-terminations not only in the CSB region of the mitochondrial insert (Figure 4A, lanes 1–7, lower part, overexposed to compensate for less labeling) but also in the vector part of the template (Figure 4A, lanes 1–7, upper part). Both the run-off and the pre-terminated products accumulated over time (Figure 4A, compare lanes 1–7 and Figure 4B-C). As before, the addition of TEFM led to a dramatic change in the ratio between full-length transcripts and shorter transcripts, and the run-off product was now by far the most dominant transcript, reaching levels 10 times higher compared to transcription without the elongation factor (Figure 4A, compare lanes 7 and 14 and Figure 4B). Even if low levels of shorter, pre-terminated transcripts were still visible in the presence of TEFM, these transcripts did not accumulate over time, but they quickly reached a maximum before stabilizing at low levels (Figure 4A, compare lanes 8–14). The abundant CSB II pre-terminated transcript is an excellent example of this effect. In the absence of TEFM, this transcript accumulated over time; however, in the presence of TEFM, it reached a peak after only 6 min (Figure 4A, lane 10) and remained stable thereafter (Figure 4A, compare lanes 8–14 and Figure 4C). The majority of all pre-terminated transcripts followed the same pattern. Additionally, we performed the same experiment on the 400 nts run-off LSP template and observed the same pattern of pre-terminated transcripts in the presence and absence of TEFM (Supplementary Figure S3C). Based on our observations, we hypothesized that POLRMT pauses at multiple sites in a given DNA sequence and is incapable of resuming transcription in the absence of TEFM, leading to transcription termination. In the presence of TEFM, these pause sites behave like true pause sites, and POLRMT is able to resume transcription, leading to more full-length transcripts. This model could explain why pre-termination sized transcripts accumulate over time in the absence of TEFM, whereas the presence of TEFM abolishes this effect. The shorter transcripts observed in the presence of TEFM would then correspond to temporarily paused polymerases destined to continue transcription, explaining why these transcripts do not accumulate over time.

Next, we investigated if TEFM could help POLRMT to reinitiate transcription of already-terminated transcripts or, alternatively, if TEFM must be present at the site and time of pausing in order to prevent premature termination. To investigate these two possibilities, we followed the accumulation of transcription products in a pulse-chase experiment. Transcription was initiated from the linearized 400 nts run-off LSP template, containing the CSB II element, in the presence of radioactive UTP. After 3 min, radiolabeling was stopped by the addition of an excess of cold UTP. To investigate the effects of TEFM on transcription termination, three different sets of samples were used: one with no TEFM (Figure 4D, lanes 1–5), one with TEFM added simultaneously with the cold UTP, i.e. at the end of the radiolabeling (Figure 4D, lanes 6–10), and one with TEFM added from the start of the transcription reactions (Figure 4D, lanes 11–15). The reactions were allowed to proceed for up to 30 min after radiolabeling had stopped. In the absence of TEFM, we observed stable levels of pre-terminated transcripts for up to 30 min after the end of radiolabeling. The addition of TEFM already at the initiation of transcription, as seen before, dramatically decreased the levels of premature transcripts (Figure 4D, lanes 11–15). In contrast, the addition of TEFM at the end of radiolabeling did not affect the levels of prematurely terminated transcripts (Figure 4D, compare lanes 1–5 and 6–10). Our results indicate that TEFM action is needed during transcript elongation, and the addition of this factor does not allow for re-initiation at the 3′-ends of terminated transcripts.

We speculated that TEFM could stabilize the transcription complex and thereby prevent termination of POLRMT-dependent transcription. To address this possibility, we used MST and monitored how TEFM affects POLRMT interactions with an RNA-primed DNA template labeled with Alexa488. In the presence of ATP, POLRMT will initiate transcription on this template and incorporate one single ATP before pausing. The stability of the elongation complex formed was measured at various POLRMT concentrations. The presence of TEFM increased the affinity of POLRMT to the DNA:RNA template and lowered the *K*_d_ from 47.8 ± 2.88 to 11.2 ± 1.00 nM (Figure 4E). We can thereby conclude that TEFM increases the stability of paused POLRMT on a DNA:RNA template, providing a possible mechanism for TEFM action. To investigate whether TEFM has an intrinsic DNA binding, we used the same DNA:RNA template and performed MST against TEFM. TEFM displayed very low affinity to this template, and we only observed interactions at very high protein concentrations, well above those used to demonstrate TEFM's ability to stimulate POLRMT interactions with the RNA-primed DNA template (Supplementary Figure S4).

Our MST results (Figures 2A and 4E) showed that POLRMT and TEFM could interact in solution, even in the absence of DNA and RNA, and that TEFM increased the POLRMT affinity to an RNA-primed DNA template. To investigate if TEFM could interact with POLRMT already at the initiation phase of transcription, we performed DNase I footprinting using an LSP template with the initiation machinery (TFAM, TFB2M and POLRMT) in the presence or absence of TEFM. In agreement with previous reports, the transcription machinery protected the TFAM binding site and regions surrounding the transcription start site (Figure 4F, compare lanes 1 and 2). Addition of TEFM made the overall footprint somewhat stronger, and we also noticed some changes in the footprinting pattern (Figure 4F, lane 3, indicated by arrows). The footprinting data thus suggest that TEFM can interact with the transcription machinery and influence its interactions with promoter DNA. TEFM on its own did not create a footprint (data not shown).

### TEFM is a POLRMT-specific factor, but it does not rely on the NTE of POLRMT

To investigate if the effect of TEFM on transcription was dependent on the direct interaction with POLRMT, we monitored the effects of TEFM on the T7 RNAP, which is similar in structure and function to its mitochondrial counterpart. We have previously demonstrated that T7 RNAP may terminate at CSB II (16), and we now investigated if TEFM could overcome this effect (Supplementary Figure S5A and B). We observed no apparent effect of TEFM on T7 transcription, neither on run-off nor on pre-terminated transcripts, indicating that the TEFM effect is POLRMT-specific.

**Figure 5.**
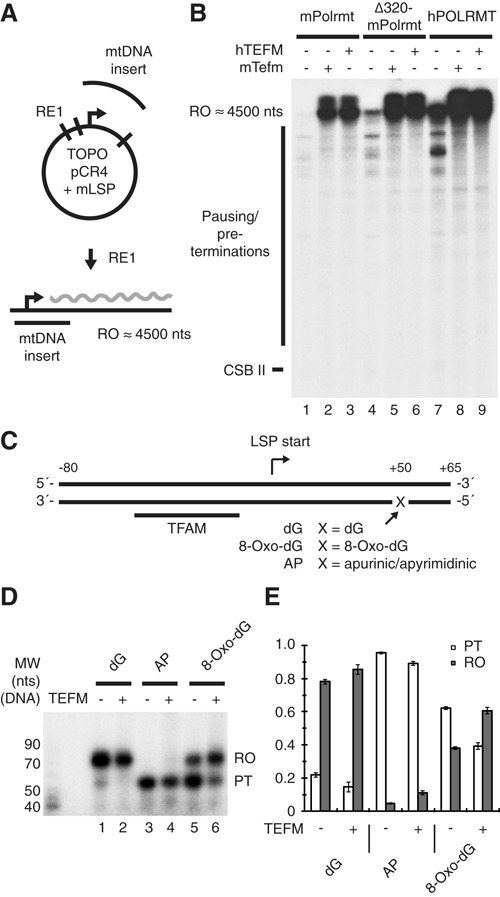
TEFM is a transcription elongation factor with cross-species function between human and mouse that do not require the NTE of POLRMT and aid the polymerase in bypass of DNA damage. (**A**) Cartoon of the template used for transcription with the mouse system, linearized to create a template with a run-off transcription product of 4500 nts. (**B**) *In vitro* transcription with the template described in panel (A) in the presence of mouse Tfam and mouse Tfb2m. Mouse Polrmt (lanes 1–3), mouse Δ320-Polrmt (lanes 4–6), or human POLRMT (lanes 7–9) was added at 20 nM. Mouse Tefm or human TEFM was added at 40 nM where indicated. Positions of run-off and CSB II-terminated transcripts are indicated. In the exposure used here, CSB II transcripts are difficult to visualize, since they are much shorter than the run-off transcription products and therefore less labeled by incorporation of radioactive UTP. (**C**) LCR-produced templates used for transcription. The templates include the TFAM binding site and the LSP promoter and generates a run-off product of 65 nts. The +50 nt, denoted as X in the figure, is either dG, 8-Oxo-dG, or an AP site in the experiment. (**D**) *In vitro* transcription on the templates described above with dG in lanes 1–2, AP in lanes 3–4 and 8-Oxo-dG in lanes 5–6. TEFM was added where indicated. The site of the 50 nts pre-termination (PT) and the 65 nt run-off (RO) are indicated. Molecular weight is indicated (Affymetrix low molecular weight 10–100 nt ladder). (**E**) Quantification of run-off and pre-terminated transcripts from panel (D). Run-off and pre-terminated transcripts are displayed as a ratio of total transcription (RO+PT). The experiment was performed in triplicate and the error bars show standard deviation.

A truncated version of the mouse Polrmt, named Δ320-Polrmt, lacking a major part of the N-terminal extension, NTE, (amino acids 1–320) can support promoter-dependent transcription *in vitro* (10). We used this truncated form of the polymerase to investigate if the NTE of POLRMT is required for TEFM to function. We performed transcription on a long mouse LSP transcription template (Figure 5A, ∼4500 nts run-off) with alternating POLRMT variants (mouse Polrmt, mouse Δ320-Polrmt or human POLRMT) in the absence or presence of mouse Tefm or human TEFM. Our results demonstrate that the polymerases from both species are fully capable of producing high levels of run-off transcripts in the presence but not in the absence of TEFM and that there are no functional differences between the mouse and the human elongation factor (Figure 5B). Additionally, the NTE of POLRMT is not required for TEFM effect *in vitro*, as transcription elongation by the Δ320-Polrmt was stimulated at a similar level to what could be observed with the full-length human and mouse polymerases. Our findings are in agreement with previous pull-down experiments, demonstrating that TEFM interacts with the catalytic domain of POLRMT (13).

### TEFM increases POLRMT bypass of 8-Oxo-dG lesions on the template strand

DNA oxidation may cause the formation of 8-Oxo-2′-deoxyguanosine (8-Oxo-dG), which, in turn, impairs POLRMT elongation. Only a small fraction of the transcribing POLRMT molecules manage to pass 8-Oxo-dG sites (19). It is known that in the nucleus, RNAP II can bypass lesions such as 8-Oxo-dG in the presence of an elongation factor, TFIIS (20). We speculated that TEFM could aid POLRMT to bypass an 8-Oxo-dG roadblock. To study this, we used LCR to synthesize templates with an 8-Oxo-dG base or an AP site (apurinic/apyrimidinic site) downstream of the LSP promoter (Figure 5C). With a deoxyguanosine (dG) at the +50 position (from the LSP start site), POLRMT produced run-off transcripts of 65 nts (Figure 5D, lane 1). In agreement with previously published results (19), a mutation of the dG at +50 to 8-Oxo-dG caused a majority of all POLRMT initiation events to terminate at this site (38% run-off and 62% pre-termination, Figure 5D lane 5 and Figure 5E). When TEFM was added to the reaction, the bypass of the 8-Oxo-dG base was increased to a majority of all initiations (61% run-off and 39% pre-termination, Figure 5D lane 6 and Figure 5E). TEFM can, therefore, aid POLRMT to transcribe past DNA lesions. For comparison, we also analyzed the effect of TEFM at AP sites. At this type of lesion, transcription was completely blocked, and TEFM could not alleviate the situation (Figure 5D, lanes 3–4 and Figure 5E). Our results show that TEFM can aid POLRMT in bypassing an 8-Oxo-dG lesion, albeit not an AP site.

## DISCUSSION

TEFM was originally identified based on sequence similarity to well-characterized transcription elongation factors. Subsequent analysis demonstrated that the knockdown of TEFM causes a decrease of promoter-distal mitochondrial transcripts, and affinity purification of TEFM also brings down POLRMT and a number of mitochondrial transcription factors. Analysis of deletion mutants indicated that TEFM interacts with the C-terminal catalytic region of POLRMT. The previously reported ability of TEFM to stimulate transcription elongation *in vitro* was relatively modest, with a mild increase in the length of transcripts on both ssDNA and dsDNA templates (13). The lack of a clear *in vitro* activity could indicate that additional factors were required for the stimulatory effect observed *in vivo*. Based on the observations presented here, we can rule out this explanation and conclude that TEFM is a factor that, on its own, can greatly stimulate POLRMT-dependent transcription. The reason for the discrepancy between the data presented here and the previous report may be trivial, as small changes in buffer conditions and purification strategies may have dramatic effects on protein activities *in vitro*. Another possible explanation for the stronger TEFM effects observed here could be the transcription system used. Whereas the previous analysis of TEFM was performed using a promoter-independent transcription system, we use the complete transcription system, including TFAM and TFB2M. As demonstrated here, TEFM interacts with POLRMT already at the promoter, and promoter-dependent transcription may therefore stimulate TEFM loading and enhance its effects on transcription elongation *in vitro*.

We find that TEFM has a strong stimulatory effect on POLRMT-dependent transcription *in vitro* and helps the polymerase to transcribe longer stretches of RNA, including regions encoding highly structured RNA (e.g. CSB II, which encodes a strong G-quadruplex-forming region). We also demonstrate that the bypassing of DNA lesions by POLRMT is increased after the addition of TEFM as pre-terminations at 8-Oxo-dG sites decrease in the presence of the elongation factor. This property of TEFM is shared with the nuclear RNAP II elongation factor TFIIS. Whether bypassing causes mutations in the RNA synthesized remains to be determined; however, the increased ability to transcribe past oxidative lesions is a clear advantage when transcribing almost the entire mitochondrial genome. Using MST, we demonstrate that TEFM interacts directly with POLRMT in the absence of other factors, such as DNA, RNA, and additional proteins. Furthermore, DNase I footprints indicate that TEFM can interact with POLRMT already during initiation of transcription. These observations may indicate that POLRMT and TEFM are engaged in a complex even when not involved in transcription, and it could thus even be debated whether TEFM is an elongation factor or rather a second subunit of the mitochondrial RNA-polymerase, much like the processivity factor POLγB, which forms a complex together with the catalytic subunit POLγA of the mitochondrial DNA-polymerase γ (1).

We find that POLRMT pauses at multiple sites in a given DNA sequence, and that in the absence of TEFM, this leads to termination of transcription. TEFM stabilizes the transcription complex, as demonstrated by its ability to increase the affinity of paused POLRMT to an RNA-primed DNA template. This stabilizing effect in turn prevents premature transcription termination and instead favors full-length transcription. The template used to monitor TEFM's effects on elongating POLRMT has previously been used in next-nucleotide incorporation assays (21) and it is similar to an elongation scaffold. How the stabilizing effect of TEFM affects transcription termination, e.g. at the MTERF1 binding site and downstream of the OriL priming site (12,22), remains to be investigated.

We have previously reported that up to two-thirds of all transcription events initiated at LSP are prematurely terminated at CSB II (2) and speculated that this effect may help to form the primers required for initiating H-strand mtDNA synthesis, as the 3′-ends of the prematurely terminated transcripts overlap with the transition points from RNA to DNA observed during mtDNA synthesis. It should, however, be noted that the replication initiation process has not yet been reconstituted *in vitro*, and additional factors and mechanisms may be essential for the regulated initiation of mtDNA synthesis in this region. Site-specific termination at CSB II is caused by G-quadruplex structures that are formed in nascent RNA when the region is transcribed and the termination is further stimulated by a short poly-dT stretch just downstream of CSB II (16). As demonstrated here, the addition of TEFM strongly reduces transcription termination at CSB II. Potentially, this observation could indicate a regulatory function for TEFM, in which the level of active TEFM governs the ratio between primer formation and full-length, productive transcription. However, we find this explanation less likely, since we here demonstrate that TEFM can stimulate transcription elongation at many different DNA sequences, suggesting that TEFM's effect cannot be limited to CSB II. We instead favor an alternative explanation. Even if TEFM inhibits transcription termination at CSB II, POLRMT still pauses at this site (as demonstrated in Figure 4A–C). Pausing may allow handover of the RNA 3′-end to POLγ and initiation of mtDNA synthesis. Another possible mechanism is that the paused RNA polymerase is released by digestion of the upstream transcript to form a primer. An enzyme that has been implicated in primer processing is the endonuclease RNase MRP (1). If this factor can work together with POLRMT in primer formation at CSB II, has to be further investigated. In the absence of TEFM, POLRMT is not as stably associated with the template and under these conditions, the strong CSB II pausing signal will cause transcription termination. Our model that the pausing is important for primer formation would explain why transcription termination in the absence of TEFM correlates closely with the RNA-to-DNA transition during initiation of H-strand DNA synthesis. In other words, TEFM will not affect the formation of the G-quadruplex in nascent RNA, but rather the ability of POLRMT to continue transcription after its formation. Also related to this effect, we have previously demonstrated that CSB II influences stable R-loop formation, which depends on a G-quadruplex structure formed between nascent RNA and the non-template DNA strand (16). Interestingly, the R-loops formed by POLRMT *in vitro* are shorter than those observed *in vivo*. In future experiments, we will investigate if TEFM can assist in R-loop elongation. We will also analyze if TEFM affects G-quadruplex formation at CSB II.

In combination, the previously published effects of TEFM on transcription elongation *in vivo* and the interactions of TEFM with POLRMT, together with the robust and dramatic effects of TEFM on transcription elongation *in vitro* reported here, establish TEFM as an essential component of the mitochondrial transcription machinery and possibly a regulator of the initiation of DNA replication. Additionally, our work will have profound effects for other researchers working in mitochondrial research and related fields, since no future studies of mitochondrial transcription, including studies of its regulation, can be performed without including TEFM in the analysis. Further genetic studies and structural work on TEFM and POLRMT are needed to gain further detailed insight into the mechanism of TEFM and transcription elongation in human mitochondria.

## SUPPLEMENTARY DATA

Supplementary Data are available at NAR Online.

SUPPLEMENTARY DATA
