# Supplementary material for: TEFM is a potent stimulator of mitochondrial transcription elongation in vitro
Source: Nucleic Acids Res. 2015 Feb 17;43(5):2615–24. doi: 10.1093/nar/gkv105 (PMC4357710; doi:10.1093/nar/gkv105)
Supplement: SUPPLEMENTARY DATA [file supp_43_5_2615__index.html]

TEFM is a potent stimulator of mitochondrial transcription elongation in vitro — SUPPLEMENTARY DATA 

# TEFM is a potent stimulator of mitochondrial transcription elongation *in vitro*

## SUPPLEMENTARY DATA

**Files in this Data Supplement:**

- SUPPLEMENTARY DATA
