# Supplementary material for: TEFM is a potent stimulator of mitochondrial transcription elongation in vitro
Source: Nucleic Acids Res. 2015 Feb 17;43(5):2615–24. doi: 10.1093/nar/gkv105 (PMC4357710; doi:10.1093/nar/gkv105)
Supplement: SUPPLEMENTARY DATA [file supp_gkv105_nar-03728-v-2014-File007.pdf]

SUPPLEMENTARY DATA

TEFM is a potent stimulator of mitochondrial transcription elongation *in vitro*

Viktor Posse[1], Saba Shahzad[2,3], Maria Falkenberg[1], B. Martin Hällberg[2,3,4], Claes M. Gustafsson[1,*]

From the [1]Department of Medical Biochemistry and Cell Biology, University of Gothenburg, SE-40530, Gothenburg, Sweden; [2]Department of Cell and Molecular Biology, Karolinska Institutet, SE-17177 Stockholm, Sweden; [3]Röntgen-Ångström-Cluster, Karolinska Institutet Outstation, Centre for Structural Systems Biology, DESY-Campus, D-22603 Hamburg, Germany; [4]European Molecular Biology Laboratory, Hamburg Unit, D-22603, Germany

[*]To whom correspondence should be addressed: Claes M. Gustafsson, Department of Medical Biochemistry and Cell Biology, University of Gothenburg, SE-40530 Gothenburg, Sweden. e-mail: claes.gustafsson@medkem.gu.se; ph. +46 31 7863826; fax. +46 31 416108

Materials and Methods

Analytic gel filtration of TEFM was performed on a Superdex 200 5/150 GL column (GE Healthcare). The column was equilibrated in 300 mM NaCl, 20 mM Hepes pH 7.5, 10% glycerol and 1 mM TCEP. The molecular weight of TEFM was calculated using five protein standards spanning the separation interval of the column (Figure S1).

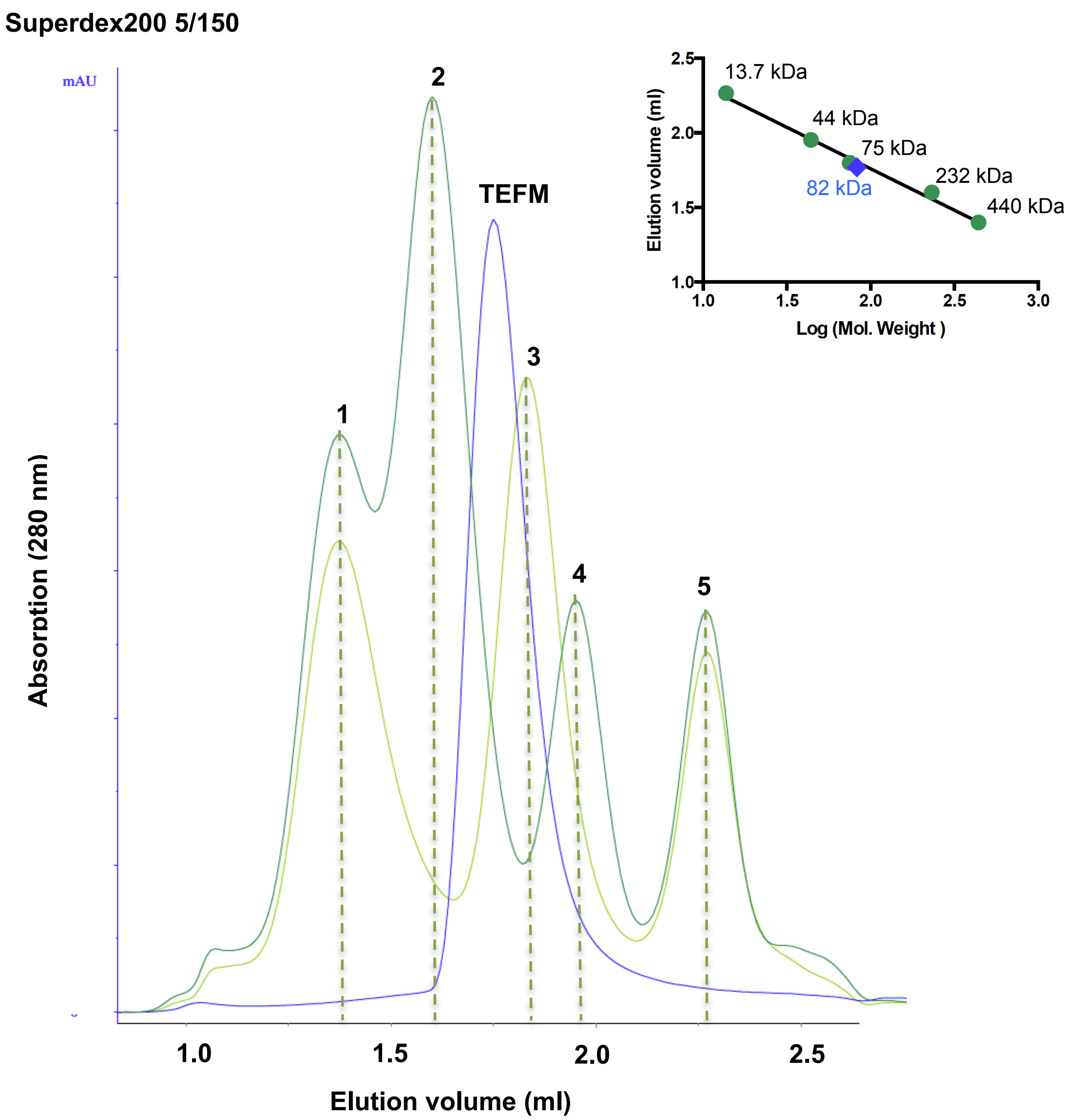
Superdex200 5/150
mAU
Absorption (280 nm)
Elution volume (ml)
1.0
1.5
2.0
2.5
1
2
3
4
5
TEFM
Elution volume (ml)
Log (Mol. Weight)
13.7 kDa
44 kDa
75 kDa
82 kDa
232 kDa
440 kDa
2.5
2.0
1.5
1.0
1.0
1.5
2.0
2.5
3.0

Figure S1. Determination of quaternary structure of TEFM. Analytical gel-filtration of TEFM was performed on a Superdex 200 5/150 GL column (GE Healthcare). Calibration of the column was performed using the following standards: 1. Ferritin (440 kDa), 2. Catalase (232 kDa), 3. Covalbumin (75 kDa), 4. Ovalbumin (44 kDa) and 5. Ribonuclease A (13.7 kDa).

Supplementary Figure S2

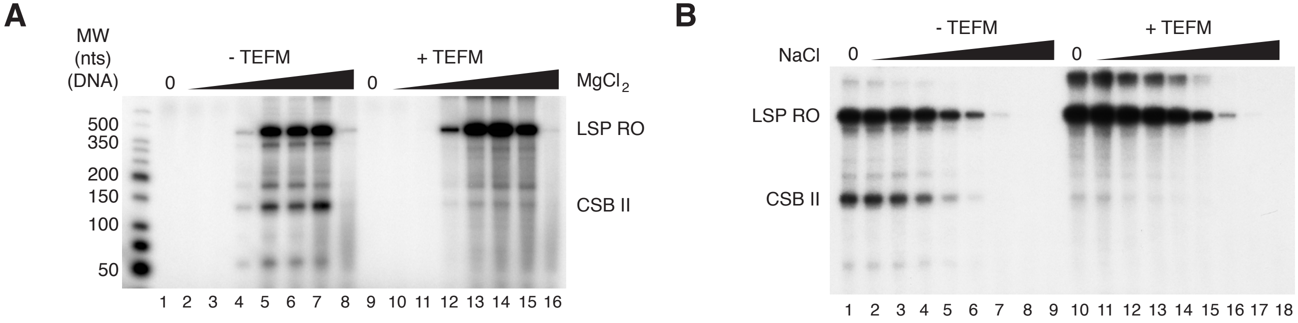

Figure S2. *In vitro* transcription from the LSP 400 nts run-off template with differing salt conditions. **(A)** $MgCl_2$ curve with increasing concentrations in lane 1-8 (in the absence of TEFM) and 9-16 (in the presence of 40 nM TEFM), $MgCl_2$ concentrations were 0, 0.5, 1, 2, 4, 8, 16 and 32 mM. LSP run-off, CSB II pre-terminated transcripts and MW marker (LMW ladder, New England Biolabs) are indicated. **(B)** NaCl curve with increasing concentrations in lane 1-9 (in the absence of TEFM) and 10-18 (in the presence of 40 nM TEFM), NaCl additions were 0, 20, 40, 60, 80, 100, 120, 140 and 160 mM. The present proteins contribute with around 10-20 mM NaCl in each reaction, salt contribution from TEFM has been compensated for. LSP run-off and CSB II pre-terminated transcripts are indicated.

Supplementary Figure S3

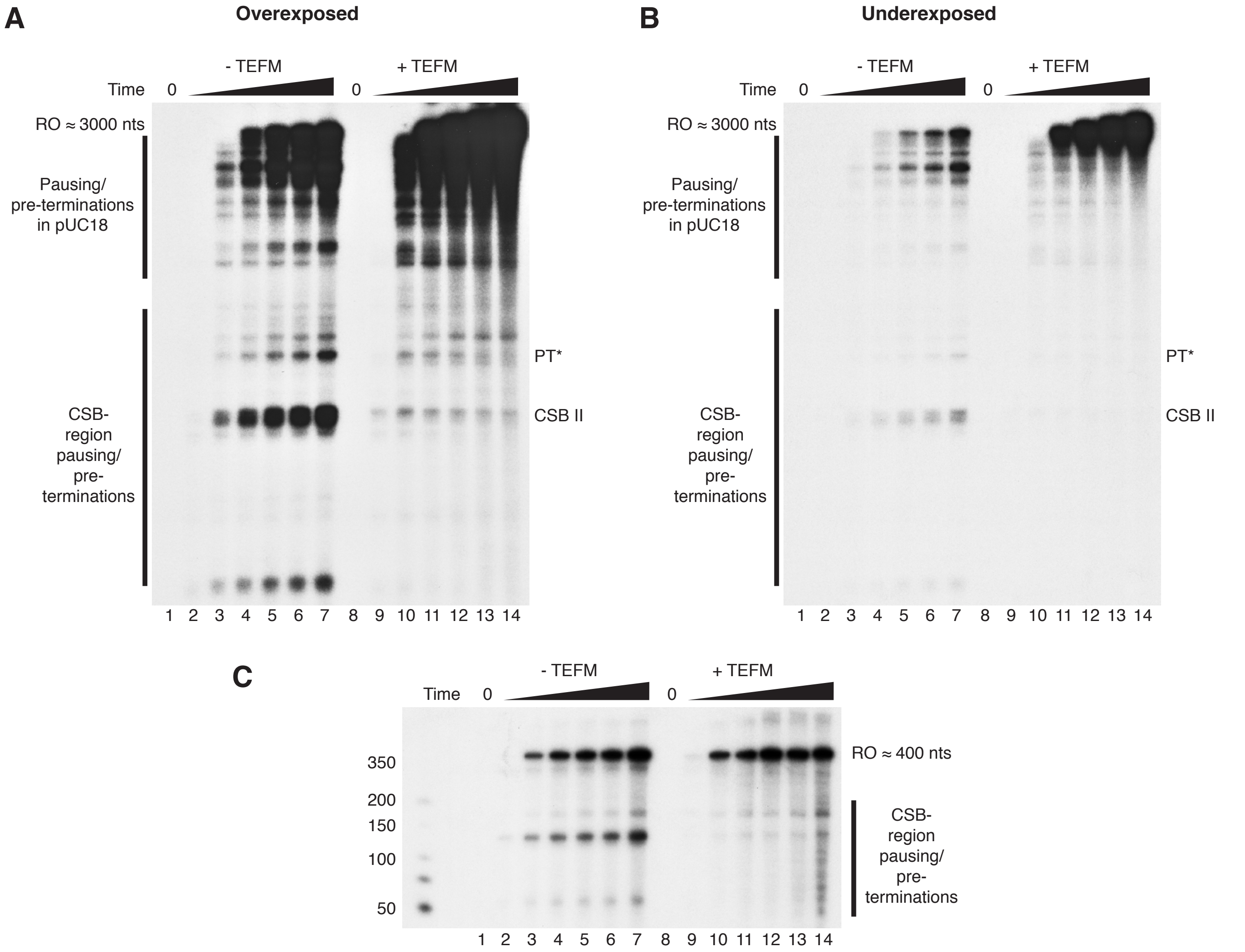

Figure S3. *In vitro* transcription from LSP on a 3000 nts **(A+B)** or a 400 nts **(C)** run-off template at different time points (0, 3, 6, 9, 12, 15 and 30 minutes) in the absence (lanes 1-7) or presence (lanes 8-14) of 40 nM TEFM. CSB region transcripts as well as run-off transcripts are indicated. For (**A**) and (**B)** the experiment is the same as in Figure 3A, but the entire films are displayed with **(A)** overexposed and **(B)** underexposed.

Supplementary Figure S4

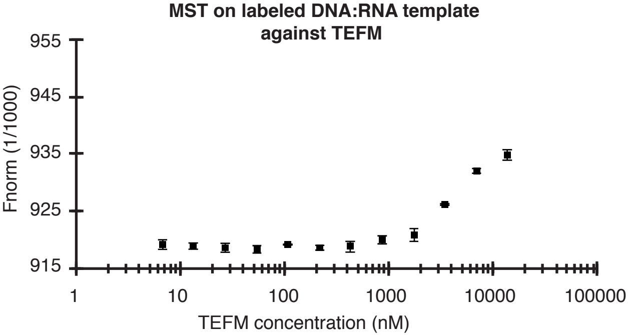

Figure S4. MST as in Figure 3E but against altering concentrations of TEFM instead of POLRMT. The binding affinity between TEFM and the DNA:RNA template was to low for K_d calculations and only at concentrations above 2000 nM any binding was observed. Since no bound state could be estimated the y values are displayed in change in thermophoresis and temperature jump, Fnorm (1/1000), instead of fraction bound.

Supplementary Figure S5

A

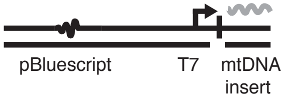

B

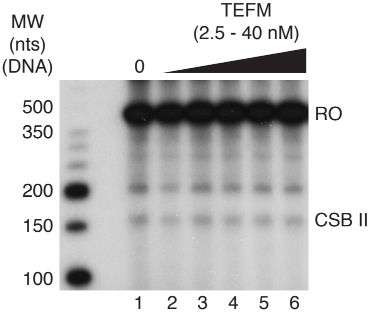

Figure S5. TEFM is a POLRMT specific transcription elongation factor. **(A)** Cartoon of the template used for T7 transcription composed of the pBluescript plasmid with an insert containing the CSB region of human mtDNA after the T7 promoter. Expected run-off product is approximately 500 nts and the CSB II product is expected to be 150 nts. **(B)** *In vitro* transcription with T7 RNAP and TEFM on the template described in panel **A**. No TEFM was added to the reaction in lane 1 and increasing concentrations of human TEFM was added in lanes 2-6 (2.5, 5, 10, 20, 40 nM respectively). The CSB II and run-off transcripts as well as molecular weight marker (LMW ladder, New England Biolabs) are indicated.
